# Concordance between Three PD-L1 Immunohistochemical Assays in Head and Neck Squamous Cell Carcinoma (HNSCC) in a Multicenter Study

**DOI:** 10.3390/diagnostics12020477

**Published:** 2022-02-13

**Authors:** Elena Guerini Rocco, Albino Eccher, Ilaria Girolami, Paolo Graziano, Gabriella Fontanini, Elena Vigliar, Giancarlo Troncone, Massimo Barberis, Patrizia Morbini, Maurizio Martini

**Affiliations:** 1 Division of Pathology, IEO, European Institute of Oncology IRCCS, 20141 Milan, Italy; elena.guerinirocco@ieo.it; 2 Department of Oncology and Hemato-Oncology, University of Milan, 20141 Milan, Italy; 3 Department of Pathology and Diagnostics, University and Hospital Trust of Verona, 37129 Verona, Italy; albino.eccher@aovr.veneto.it; 4 Division of Pathology, Central Hospital Bolzano, 39100 Bolzano, Italy; ilaria.girolami@sabes.it; 5 Unit of Pathology, Fondazione IRCCS Casa Sollievo Della Sofferenza, San Giovanni Rotondo, 71013 Foggia, Italy; p.graziano@operapadrepio.it; 6 Department of Surgical, Medical, Molecular Pathology and Critical Area, University of Pisa, 56126 Pisa, Italy; gabriella.fontanini@unipi.it; 7 Department of Public Health, University of Naples “Federico II”, 80125 Naples, Italy; elena.vigliar@unina.it (E.V.); giancarlo.troncone@unina.it (G.T.); 8 Clinical Unit of Oncogenomics, IEO, European Institute of Oncology, IRCCS, 20141 Milano, Italy; massimo.barberis@ieo.it; 9 Unit of Pathology, Department of Molecular Medicine, Università di Pavia, Foundation IRCCS Polilcinico San Matteo, 27100 Pavia, Italy; 10 Department of Human Pathology of the Adult and Developmental Age “Gaetano Barresi”, University of Messina, Via Consolare Valeria n. 1, 90128 Messina, Italy

**Keywords:** PD-L1, head and neck squamous carcinoma, 22C3 and SP263 assays

## Abstract

The introduction of immunotherapy targeting the programmed death-1 (PD-1)/programmed death-ligand-1 (PD-L1) axis has represented a turning point in the treatment of HNSCC. Harmonization studies comparing the different antibodies and immunohistochemistry platforms available for the evaluation of PD-L1 expression with Combined Positive Score (CPS) in HNSCC are strongly required. Tissue microarrays (TMA) constructed from formalin-fixed, paraffin-embedded (FFPE) tissue blocks of HNSCC tumor were stained with two commercial in-vitro diagnostic (IVD) PD-L1 immunohistochemical assays (22C3 pharmDx on Autostainer Link48 and Omnis platforms, and SP263) and were reviewed by seven trained pathologists to assess CPS. We found a very similar distribution for PD-L1 expression between 22C3 pharmDx assay with both platforms and SP263 assay and a strong significant correlation between the two assays in different platforms (*p* < 0.0001). The interobserver reliability among pathologists for the continuous scores of CPS with intraclass correlation coefficient (ICC) and the correlation between the two assays were both good. Moreover, the agreement rate between assays was high at all cut-offs, while the kappa values were from substantial to almost perfect. These data suggest the interchangeability of the two antibodies and of the different immunohistochemical platforms in the selection of patients with HNSCC for immunotherapy.

## 1. Introduction

With approximately 880,000 new patients each year, head and neck squamous cell carcinoma (HNSCC) represents the sixth-most common type of cancer worldwide [1]. Primary resection followed by radio-chemotherapy is at present the standard of care for locally advanced HNSCCNevertheless, the 5-year overall survival in advanced stage HNSCC has not improved beyond 50–65% over the past three decades [2,3]. The introduction of immunotherapy targeting the programmed death-1 (PD-1)/programmed death-ligand-1 (PD-L1) axis has represented a turning point in the therapy of HNSCC. The randomized phase 3 trial KEYNOTE-040 compared pembrolizumab versus standard-of-care therapy in patients with recurrent or metastatic (R/M) HNSCC. Patients were stratified according to PD-L1 expression levels, assessed as tumor proportion score (TPS) on immunohistochemistry (IHC), using the commercial 22C3 pharmDx assay [4]. KEYNOTE-040 trial results led to the approval of pembrolizumab as a second-line treatment in patients with TPS ≥ 50%. The subsequent KEYNOTE-048 phase 3 trial compared pembrolizumab alone or in combination with platinum and 5-fluorouracil-based chemotherapy [5]. In this trial the combined positive score (CPS), considering both PD-L1-positive tumor cells and tumor-infiltrating leukocytes (TIL), was used to assess PD-L1 expression levels [5]. The results of Keynote 048 study led the Federal Drug Administration (FDA) to approve pembrolizumab as first-line therapy in patients with recurrent/metastatic HNSCC in 2019 [6]. Pembrolizumab was approved in monotherapy for patients with CPS ≥ 1 assessed with an IHC test approved by FDA-, and in combination with platinum and fluorouracil regardless of PD-L1 status. In 2020, the European Medicines Agency (EMA) approved the use of pembrolizumab as first-line treatment for R/M HNSCC in patients with CPS ≥ 1 in monotherapy or in combination with chemotherapy, and as second-line therapy for tumors with TPS ≥ 50%, regardless of the test (antibody and IHC platform) used [7]. Post hoc analysis of KEYNOTE 040 trial results later demonstrated that CPS and TPS can be used interchangeably at or above 50% cut-off, while CPS sensitivity is higher than TPS at lower cutoffs [8]. Moreover, an increased benefit from therapy was seen in tumors with CPS ≥ 20 [5]. 

An accessible, reproducible, and robust IHC test for the evaluation of PD-L1 expression in terms of CPS is therefore crucial to guide therapy decisions in patients with HNSCC. However, technical and biological issues still hamper CPS assessment by pathologists in the clinical practice [9,10]. First, several antibody clones and platforms have been developed for the immunohistochemical analysis of PD-L1 expression. PD-L1 22C3 pharmDx kit used on the Autostainer Link48 platform is the only FDA-approved companion diagnostic for single-agent pembrolizumab treatment [6]; no specific assay has been designated by EMA [7]. PD-L1 22C3 pharmDx and Ventana PD-L1 (SP263) IHC tests showed a high correlation in non-small cell lung cancer (NSCLC), where TPS evaluation is required [11,12]. In HNSCC, studies comparing different assays for CPS evaluation are still scarce [10,13]. Moreover, CPS assessment seems more complex and perhaps less intuitive than TPS, because it takes into account the staining of subsets of tumor-associated immune cells besides tumor cells. Not surprisingly, specific training is required for CPS assessment, and variable concordance among pathologists has been reported [10,14,15]. Finally, intra- and inter-tumor heterogeneity of PD-L1 expression has been described in HNSCC as well as in other types of tumors, which may impact the evaluation of CPS, especially in small biopsy specimens [10,16].

In this study, tissue microarrays (TMA) constructed from formalin-fixed, paraffin-embedded (FFPE) tissue blocks of HNSCC primary tumor and/or lymph node metastasis, and stained with different commercial IVD PD-L1 immunohistochemical assays were reviewed by a pool of trained pathologists to assess CPS. The study aimed to: (i) compare CPS on sections stained with the different IVD PD-L1 immunohistochemical assays; (ii) assess the agreement in CPS evaluation among trained pathologists.

## 2. Materials and Methods

### 2.1. Sample Collection and Preparation

Forty-seven FFPE tissue blocks of primary tumor and/or lymph node metastasis from 40 patients who underwent surgical resection of HNSCC between 2020 and 2021 were retrospectively collected for this study by three participating pathologists (EGR, MM, PM). Samples had been fixed in 10% buffered formalin for 12 to 48 h and embedded in paraffin. Hematoxylin and eosin-stained sections from each sample were reviewed and representative areas including tumor and intra/peritumoral stroma were selected for the preparation of TMAs. Two to four cores (total 128 cores; median 3.2/case) were obtained from different areas of each block with a 1-mm puncher on a semi-automatic tissue microarrayer (Galileo TMA, CK4500B, Integrated Systems Engineering, Milan, Italy) and transferred to two recipient TMA blocks, each containing a total of 50 cores and two reference cores of human placental tissue. Three-micron-thick sections cut from TMA blocks were used for hematoxylin and eosin stain (H&E) and immunohistochemistry. Patients who underwent neoadjuvant chemotherapy and/or radiotherapy were excluded from this study. HPV status in oropharyngeal SCC and in metastases was assessed by CINtec p16 Histology assay (Roche, Milan, Italy) with strong and diffuse nuclear and cytoplasmic staining in at least 70% of cells used as the cut-point for positivity [15]. All patient data were collected anonymously and written informed consent, as part of the routine diagnosis and treatment procedures, was obtained from patients or their guardians according to the Declaration of Helsinki and the study adhered to Good Clinical Practice guidelines.

### 2.2. Immunohistochemical Assays and Evaluation

Immunohistochemistry was performed on consecutive sections cut from TMA blocks using PD-L1 IHC 22C3 pharmDx assay for the Dako Autostainer Link 48 (22C3-Autostainer) and Dako Omnis (22C3-Omnis) platforms (Agilent Technologies, Santa Clara, CA, USA), and Ventana PD-L1 (SP263) assay for Ventana BenchMark platform (Ventana Medical Systems, Tucson, AZ, USA), according to the manufacturer’s instructions [17,18,19]. Reference cores of human placental tissue included in each TMA were used as a positive control. Immunohistochemical analysis was centralized and performed at Molecular Pathology Laboratories of Istituto Europeo di Oncologia (22C3—Autostainer), University of Pavia (22C3-Omnis) and University of Verona (SP263). PD-L1 control slides from 22C3 pharmDx (containing sections of two pelleted, formalin-fixed paraffin-embedded cell lines: NCI-H226 with moderate PD-L1 protein expression and MCF-7 with negative PD-L1 protein expression) were used as positive and negative control for both antibodies (22C3 and SP263). All stained slides were digitized with an Aperio CS2 instrument (Leica Biosystem, Milan, Italy) at ×40 magnification, uploaded on a shared web platform provided by Nikon, and viewed with NDP.view2 software by seven head and neck pathologists specifically trained and certified in CPS assessment from all participating Centers. CPS was determined as the number of PD-L1 positive tumor cells, lymphocytes, and macrophages divided by the total number of viable tumor cells, multiplied by 100. Any perceptible and convincing partial or complete linear membrane staining of viable tumor cells that was recognized as distinct from cytoplasmic staining at ×20 magnification was considered as positive and included in the scoring. Likewise, any membrane and/or cytoplasmic staining of mononuclear inflammatory cells within tumor nests and/or adjacent supporting stroma was considered as positive and included in the CPS numerator. Neutrophils, eosinophils, plasma cells, and immune cells associated with tumor, in situ carcinoma, normal structures, or ulcers were excluded from the CPS score. CPS was expressed as continuous values comprised between <1 and 100. Each countable core section contained at least 100 viable HNSCC cells. All pathologists had received appropriate training for CPS score evaluation in HNSCC and were blinded to clinical information as well as to the evaluations of other pathologists. 

### 2.3. Statistical Analysis

CPS per patient were obtained as the mean of all TMA cores obtained from the same patient. To test inter-observer agreement in the evaluation of CPS expressed as continuous values, intraclass correlation coefficient (ICC) was calculated for each assay on TMAs. To test inter-observer agreement at relevant clinical CPS cut-offs of <1, 1–20, >20, Fleiss’s kappa for multiple raters was calculated at each cut-off and for the overall values of the cut-off categories for each assay on TMAs. To test inter-assay reproducibility, comparison of CPS expressed by each observer in terms of both continuous values and cut-offs among pairs of assays (22C3-Autostainer vs. 22C3-Omnis, 22C3-Autostainer vs. SP263, and 22C3-Omnis vs. SP263) was performed, and expressed respectively with ICC and Cohen’s kappa. Statistical analyses were performed using Microsoft Excel 2013, IBM SPSS Statistics for Windows, Version 25.0 (IBM Corp: Armonk, NY, USA) and R software version 4.0.0 (R Foundation for Statistical Computing, Vienna, Austria).

## 3. Results

### 3.1. Patient Characteristics and PD-L1 Staining by 22C3 Pharma Dx and SP263 Assays

The sample analyzed included 40 tissue blocks from surgical specimens of HNSCCs collected between 2020 and 2021. Main clinicopathologic characteristics of our cohort are reported in Table 1. 

**Figure 1:**
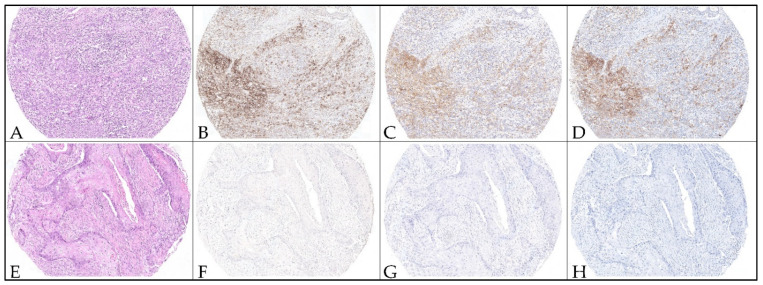
Representative micrographs of PD-L1 immunohistochemistry staining with different assays on TMA cores from head and neck squamous cell carcinoma. Hematoxylin and eosin-stained cores (**A**,**E**) with positive and negative PD-L1 expression. TMA cores from a case showing high CPS score values (CPS > 1) using the 22C3 pharmDx assay on Autostainer Link48 platform (**B**), 22C3 assay on Omnis platform (**C**), and SP263 assay (**D**). TMA cores from a case showing negative CPS score value (CPS < 1) using the 22C3 pharmDx assay on Autostainer Link48 platform (**F**), 22C3 assay on Omnis platform (**G**), and SP263 assay (**H**). Original magnification 100×.

Mean age at the time of diagnosis was 63 years; 65% of patients were males. Thirty-one patients (77.5%) had metastatic disease and nine (22.5%) had unresectable/recurrent neoplasms. Tumor originated from the oral cavity in 18 out of 40 patients (45%) (including the oropharynx), hypopharynx in 15 (37.5%), larynx in five (12.5%); two (5%) had metastatic localization. Seven out of 20 (35%; including 18 oral SCC and two neck nodal metastases) were positive for p16. PD-L1 IHC was performed using the 22C3 pharmDx, on two different platforms, and the SP263 assays and the CPS per patient were calculated by taking the mean of all TMA cores (Figure 1). 

**Figure 2:**
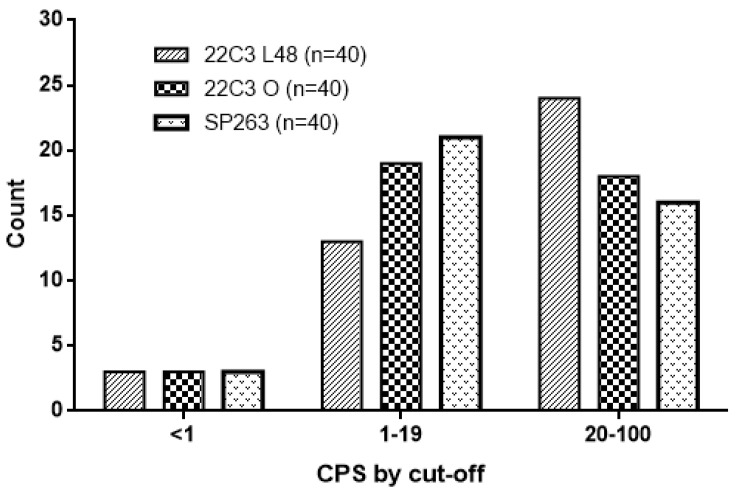
The figure shows the distribution of PD-L1 expression (as CPS) of the 22C3 pharmaDx, on Dako Autostainer link48 (L48) and Dako Omnis (O), and of SP263 assay for the different CPS cut-offs (<1; between 1 and 20 and >20).

When we considered the cut-off of ≥1, 37 cases (92.5%) had a positive CPS score with both 22C3 pharmaDx tests and SP263 assays. CPS values between 1 and 20 were scored respectively in 13/40 (32.5%) cases with 22C3-Autostainer, in 19/40 (47.5%) cases with 22C3-Omnis and in 21/40 (52.5%) cases with SP263 assay. CPS values ≥ 20 were diagnosed in 24 out of 40 (60%) cases with 22C3 -Autostainer, in 18 out of 40 (45%) cases with 22C3-Omnis and in 16 out of 40 (40%) cases with SP263 assay (Table 1). Three samples (7.5%) had a CPS < 1 in all cores with both assays on the three platforms. The distribution of CPS is shown in Figure 2. 

**Figure 3:**
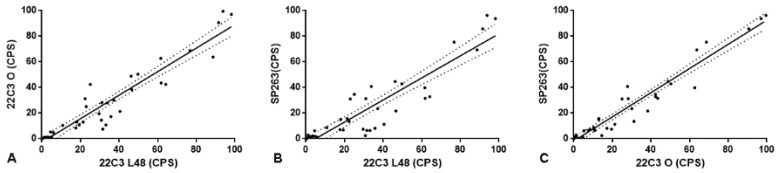
(**A**): the figure shows the direct and significant correlation between CPS evaluated with 22C3 pharmaDx on Dako Autostainer link48 and Dako Omnis (O; Spearman r = 0.900 *p* < 0.0001). (**B**): the figure shows the direct and significant correlation between CPS evaluated with 22C3 pharmaDx on Dako Autostainer link48 and SP263 assay (Spearman r = 0.893 *p* < 0.0001). (**C**): the figure shows the direct and significant correlation between CPS evaluated with 22C3 pharmaDx on Dako Omnis (O) and SP263 assay (Spearman r = 0.935; *p* < 0.0001).

The 22C3 and SP263 assays showed a similar distribution of CPS values, although the number of cases with CPS ≥ 20 was slightly higher with 22C3-Autostainer in comparison to SP263 assay. Comparing the CPS values obtained with 22C3-Autostainer and those obtained with 22C3-Omnis, we found a significant correlation between the two platforms (Spearman r = 0.900 *p* < 0.0001; Figure 3, panel A). A significant correlation was also observed when we compare the CPS values obtained with both 22C3-Autostainer and 22C3-Omnis, with those obtained with the SP263 assay (Spearman r = 0.893 *p* < 0.0001; Figure 3, panel B; Spearman r = 0.935; *p* < 0.0001; Figure 3, panel C, respectively). 

### 3.2. Concordance among Pathologists

The inter-rater correlation was always good (>0.500) for all the assays (Table 2). The highest values were reached in the evaluation of CPS with 22C3—Omnis, with ICC of 0.938 (95% CI of 0.914–0.957), and with SP263 assays, with ICC of 0.930 (95% CI of 0.899–0.953) while the lowest values was reported with 22C3—Autostainer, with ICC of 0.914 (95% CI 0.881–0.941). 

Concerning the agreement at clinically relevant cutoffs, the Fleiss kappa among raters was from substantial to almost perfect, ranging from 0.748 to 0.895 with 22C3—Autostainer, from 0.675 to 0.848 with 22C3—Omnis and from 0.720 to 0.880 using SP263. For all assays, the agreement was lowest for the cut-off class CPS 1–20 and slightly higher for the cut-off class CPS > 20 and CPS < 1.

### 3.3. Concordance among Assays

The correlation between couples of assays was always good (>0.500) for all the observers (Table 2). The highest concordance values were reached in the comparison between 22C3—Omnis and SP263 assays, with ICC ranging from 0.874 to 0.993, while the lowest values were reported in the comparison between 22C3—Autostainer and SP263 assays, with ICC ranging from 0.532 to 0.807.

Concerning the agreement at clinically relevant cutoffs, the Cohen’s kappa in the TMA set ranged from moderate to strong (0.68–0.82) in the comparison between 22C3—Autostainer and 22C3—Omnis, from weak to moderate (0.52–0.69) in the comparison between assay 22C3—Autostainer and SP263, and from moderate to strong (0.64–0.80) in the comparison between 22C3—Omnis and SP263.

## 4. Discussion

The approval of pembrolizumab for treatment of recurrent/metastatic HNSCC has challenged the laboratories of pathology dealing with head and neck oncology to assess a predictive test for the first time. As for most other immune check-point inhibitors, the registration of pembrolizumab by FDA was associated with a specific diagnostic companion test, scoring system and cut-off values, while subsequent approval by EMA and UK’s NICE did not specify the type of test that should be used [20]. Treasuring the previous experience gained with PD-L1 testing in NSCLC, a series of crucial issues required investigation in the setting of HNSCC, including inter-pathologist reproducibility in the assessment of CPS. Moreover, the presence of different commercial platforms and antibody clones for the immunohistochemical staining of PD-L1, in the absence of a prescribed companion test, questioned the inter-platform and inter-clone reproducibility of CPS results. Our study was designed to explore inter-observer and inter-platform concordance using the two standardized PD-L1 assays most used in pathology laboratories in our country. We compared the 22C3 PharmDx test optimized for the Dako Autostainer Link48 platform, appointed by FDA as the companion diagnostic test for the selection of patients to be treated with pembrolizumab, with the widely diffuse, but not specifically validated, SP263 anti-PD-L1 antibody clone standardized for the use with the Ventana Benchmark platform. The observation that several laboratories are updating to the Dako Omnis platform prompted us also to compare the performance of PharmDx assays standardized for Dako Omnis with that of the reference test.

While relevant literature data support a high inter-observer agreement for TPS evaluation in NSCLC [11,12], it is reasonable to expect lower agreement for CPS, due to the increased complexity of recognizing and quantifying PD-L1-expressing TIL subsets alongside neoplastic epithelial cells. Here, we reported a very good concordance among pathologists both for continuous CPS values and at relevant cut-offs (≥1, >20). Inter-observer concordance rates did not significantly differ for assays performed on the three platforms, confirming that all provide good quality staining which do not pose relevant issues of interpretation. Of note, with the three platforms the lowest kappa values were concordantly observed in the intermediate CPS 1–20 class, while inter-observer agreement was very high for the CPS < 1/≥ 1 cutoff that directly affects treatment decision.

This is the second study reporting high inter-observer agreement among several pathologists (seven in the present study, 10 in the previous one) for CPS evaluation in HNSCC. In both studies, pathologists had received dedicated training in the assessment of CPS in HNSCC [15]. These results are strong evidence that training programs to update pathologists on new criteria to be applied for in situ biomarker evaluation are highly effective [9,14].

Inter-platform and inter-clone agreement for TPS evaluation in NSLC has also been widely investigated [11,12], while only few studies have been published so far focusing on CPS in HNSCC. A recent study designed to compare 22C3-Autostainer Link48 and SP263-Benchmark performance on 43 whole tissue sections reported high inter-platform concordance rates [15], and confirmed previous limited observations in surgical samples [21], thus supporting the possibility of using the two tests interchangeably. Although our study was conducted on tissue-microarray (TMA) and not on whole tissue sections, our results are in line with the previous observations. One previous study [22], reporting low concordance between the two Dako platforms was performed using 22C3 antibody as LTD on Dako Omnis before the PharmDX kit had been optimized for use on this platform. We providednewevidence that validates the PharmDX assay optimized for use on Dako Omnis platform in comparison to Dako Autostainer link48, in the light of the ongoing shift between the two platforms. Overall, our results also suggest that the three platforms are equally robust at relevant CPS cut-offs and may be used interchangeably according to availability in each laboratory. 

In contrast to our evidence, a large TMA-based study [13], comprising 143 cases, reported considerable differences between the two standardized assay 22C3 on Autostainer Link48 platform and SP263 on Benchmark platform, both as continuous values and at relevant cut-offs of positivity, with SP263 providing higher CPS scores. Despite the lower number of patients analyzed, our results showing high levels of concordance have several strengths. The fact that TMA samples had been obtained from different institutions and stained in three independent laboratories obtaining high concordance rates indicates that PD-L1 IVD tests are robust with respect to preanalytical variables. Moreover, the high inter-observer agreement between more pathologist trained and certified for the CPS evaluation adds further value to our results. The remarkably different levels of concordance could thus depend on sampling or analytical variables not taken into account.

The most relevant limitation of the present study is the choice of using TMA instead of whole tissue sections, for practical reasons related to the process of sample collection and sharing. TMA use for semiquantitative analysis such as CPS evaluation could be positively biased by the limited tissue surface to be examined, and provide lower inter-observer variability, thus poorly representing “real life” examination of surgical sample slides. However, the rate of inter-observer agreement reported in our study was comparable to that of a recent CPS study performed on whole HNSCC tissue sections [15]. This observation confirms the utility of TMAs in studies with elevated number of samples/observers and suggests that the optimal core number to assure representativity of histological samples could be three cores/sample. 

Another limitation, shared by all studies published so far, is the lack of correlation with tumor site and previous treatment(s). It is reasonable to expect that tumors originating in distinct anatomical subsites of the head and neck region will have a different interaction with the immune microenvironment, and that the treatment history of the disease, in particular radio and chemotherapy, may modulate the immune check-point activation. In fact, several studies seem to demonstrate a role of cisplatin-based therapeutic regimens in the induction of PD-L1 expression in HNSCC [23,24,25], while other studies have recently highlighted that radiotherapy could deeply affect the tumor microenvironment and the immune response against tumor also influencing the expression of PD-L1 (abscopal effect) [26]. 

Finally, there are two other limitations: we analyzed a low number of cases with a relative under-representation of some specific head and neck districts and of metastatic sites, and pathologists assessed the cases remotely using their own personal workstations. This implies a potential evaluation bias related to lack of standardization of viewing displays and network bandwidth.

## 5. Conclusions

Our data demonstrates the substantial interchangeability between SP263 assay and 22C3 pharmaDx and three different immunohistochemical platforms in the PD-L1 evaluation of HNSCC patients. However, further studies in other independent cohorts are needed to confirm our data and definitively support the harmonization of the different PD-L1 assays on TMA and whole sections samples.

## Figures and Tables

**Table 1:** Patient characteristics.

	*n = 40*
*Age, mean (±SD)*	63 (7.7)
*Gender, n (%)*	
*Male*	26 (65)
*Female*	14 (35)
*Stage, n (%)*	
*metastatic*	31 (77.5)
*unresectable recurrent*	9 (22.5)
*Tumor location, n (%)*	
*Oropharynx*	18 (45)
*Hypopharynx*	15 (37.5)
*Larynx*	5 (12.5)
*Metastatic sites*	2 (5)
*HPV status (p16), n (%)*	
*Positive*	7 (35)
*Negative*	13 (65)
*PD-L1 expression 22C3 Auto L48 n (%)*	
*<1*	3 (7.5)
*1–<20*	13 (32.5)
*≥20*	24 (60)
*PD-L1 expression 22C3 Omnis, n (%)*	
*<1*	3 (7.5)
*1–<20*	19 (47.5)
*≥20*	18 (45)
*PD-L1 expression SP263, n (%)*	
*<1*	3 (7.5)
*1–<20*	21 (52.5)
*≥20*	16 (40)

**Table 2:** Measure of agreement.

**Concordance among Raters—ICC**	*22C3 Omnis*	0.938 (CI 0.914 to 0.957)
	*SP263*	0.930 (CI 0.899 to 0.953)
	*22C3 Autostainer*	0.914 (CI 0.881 to 0.941)
**Concordance among Raters for Cut-Off Categories—Fleiss’ Kappa**	*22C3 Omnis*	Range 0.675–0.848
	*SP263*	Range 0.720–0.880
	*22C3 Autostainer*	Range 0.748–0.895
**Concordance among Assays—ICC**	*22C3 Omnis vs. SP263*	Range 0.874–0.993
	*SP263 vs. 22C3 Autostainer*	Range 0.532–0.807
	*22C3 Autostainer vs. 22C3 Omnis*	Range 0.686–0.924
**Concordance among Assays for Cut-Off Categories—Cohen’s Kappa for Single Rater**	*22C3 Omnis vs. SP263*	Range 0.642–0.796
	*SP263 vs. 22C3 Autostainer*	Range 0.522–0.687
	*22C3 Autostainer vs. 22C3 Omnis*	Range 0.681–0.823

## Data Availability

The datasets used and/or analyzed during the current study are available from the corresponding authors on reasonable request.
